# AptaSUITE: A Full-Featured Bioinformatics Framework for the Comprehensive Analysis of Aptamers from HT-SELEX Experiments

**DOI:** 10.1016/j.omtn.2018.04.006

**Published:** 2018-04-22

**Authors:** Jan Hoinka, Rolf Backofen, Teresa M. Przytycka

**Affiliations:** 1 National Center of Biotechnology Information, National Library of Medicine, NIH, Bethesda, MD 20894, USA; 2 Bioinformatics Group, Department of Computer Science, University of Freiburg, Freiburg 79110, Germany

**Figure:**
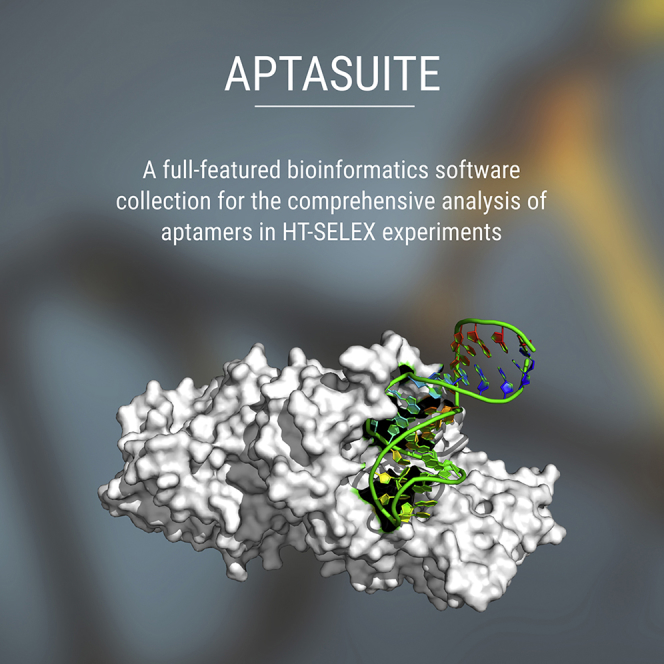

## Main Text

**To the editor:**

The capability of producing and efficiently processing big data has revolutionized virtually every field of science and technology and has enabled the analysis of experimental results at unprecedented resolutions. This trend is also evidenced in the rapid emergence, and subsequent field-wide adoption, of the high-throughput systematic evolution of ligands by exponential enrichment (HT-SELEX) protocol in the study of *in vitro* selection.^1^ HT-SELEX extends the traditional SELEX protocol, aimed at the generation of high-affinity and specificity oligonucleotides known as aptamers against a molecular target of interest, by coupling this technology with HT sequencing. Selection is typically performed in iterations and consists of incubating an initially random pool of sequences with the target, followed by the partitioning, and subsequent removal, of non-affine species while amplifying the remaining pool to form the input to the next cycle as well as the source material for sequencing. The resulting sequencing data, consisting of a representative sample of the pool composition after each round of selection, are consequently analyzed *in silico* through dedicated algorithmic approaches, whereby aptamers predicted to possess the desired application-specific properties are typically subjected to further *in vitro* verification and post-processing.

Notably, in order to guarantee an efficient and accurate *in silico* pipeline, these computational methods must be carefully designed to maximize efficiency while scaling well vertically (guaranteeing a proportional reduction in computation time with a growing number of available processing units and memory) as well as with increasing data volume. Ideally, such tools would additionally require a low learning curve for experimentalists, be platform-independent, and provide integrated means of storing and retrieving, interacting with, and visualizing aptamer-related information.

Indeed, over the past decade, typical HT-SELEX datasets have grown 200-fold from 10,000–100,000 reads per selection cycle to current sizes of routinely over 20 million reads per round. This development constitutes an emerging barrier for many well-established algorithmic tools devised before the big data revolution but which are still actively used in RNA bioinformatics analysis pipelines. Prominent examples include clustering sequence species into aptamer families related to each other by sequence similarity^2^, ^3^, ^4^ and the elucidation of shared motifs in primary and/or secondary structure evolving throughout the selection. Both analysis tasks are well established for small-scale datasets but rapidly become computationally intractable with increasing data volume when performed with traditional methods.^5^, ^6^ More complex approaches, designed for scalable, HT data processing on multi-core environments, as found in data centers and cloud environments, typically require expert knowledge to set up a sensible pipeline and may depend on numerous, potentially non-portable, third-party software packages, increasing the burden of long term maintainability.^7^, ^8^, ^9^ In addition, the resulting processed data are predominantly output in pure text format or stored in relational databases, adding to the stack of challenges in efficiently interpreting the results. Finally, while undoubtedly being of great utility, web-based (and therefore graphical) solutions backed by cloud services such as the Galaxy project^10^, ^11^ are limited in their flexibility of visualizing and interacting with vast amounts of data as they must adhere to the constraints imposed by current web browsers and technologies.

**Figure 1:**
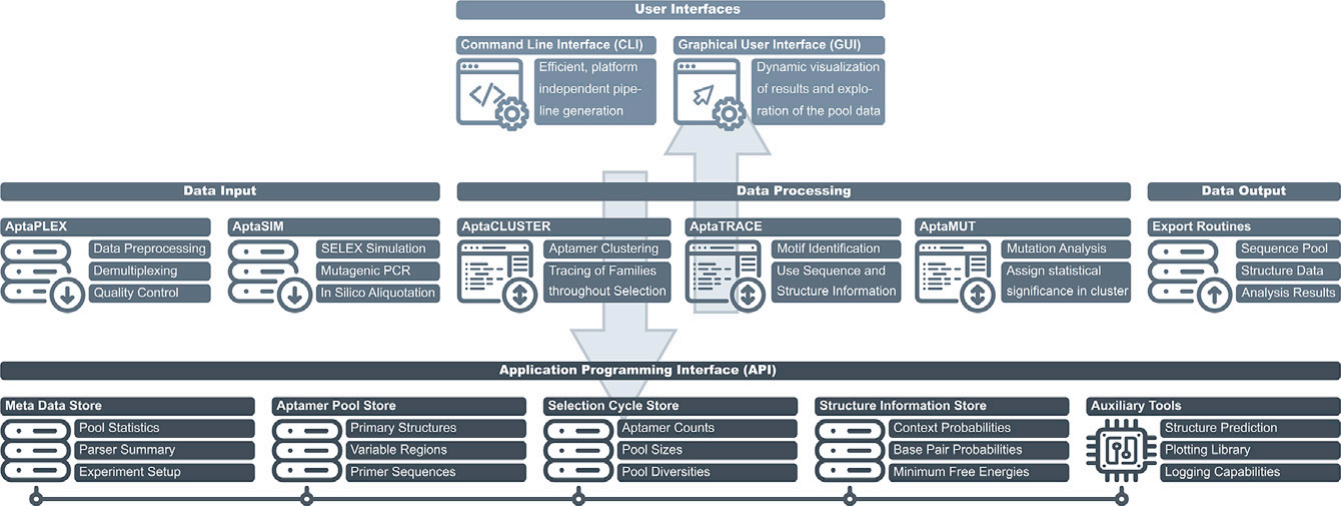
The Modularized Architecture of AptaSuite Diagram depicting the programmatic architecture of AptaSuite. Core libraries for the storage, retrieval, and manipulation of aptamers are accessed through a well-defined API which, in turn, serves data to and accepts data from the algorithms responsible for input, processing, and output of aptamers. Core libraries include efficient solutions for storing primary and secondary structure information regarding the accepted aptamers, a digital representation of the performed selection by storing the experimental setup, as well as information about the performed selection cycles and auxiliary tools, such as secondary structure prediction algorithms, which have been ported to Java to maintain platform independence. The software layer currently features AptaPLEX, a multithreaded demultiplexer for HT-SELEX data; AptaSIM, aimed at realistically simulating the selection dynamics of SELEX experiments; AptaCLUSTER for the efficient determination of aptamer families; AptaMUT, tailored to the identification of mutants with improved binding affinity; and AptaTRACE, an efficient algorithm for sequence-structure motif elucidation utilizing the entirety of the available aptamer pools. Finally, each computational method is accessible either from command line or through the graphical user interface.

To address these issues, we have developed AptaSuite, a full-featured, open source, and platform-independent software collection for the comprehensive analysis of HT-SELEX experiments. In stark contrast to previous methods, each implementing their individual and frequently rudimentary data workflow, AptaSuite provides a unified and robust framework for managing aptamer-related data and leverages this framework to serve the required data in a standardized manner to any particular algorithm built with the software. In its core, AptaSuite consists of a collection of carefully designed APIs (application programming interfaces) and corresponding reference implementations for facilitating input, output, and manipulation of aptamer data (such as sequences, aptamer counts in individual selection cycles, structure information, and more). On top of this powerful core library, a number of previously published approaches^8^, ^12^, ^13^, ^14^, ^15^ have been implemented from scratch and are now combined into this uniform, easy-to-use framework (see Figure 1). In particular, the selected methods constitute well-established approaches to analyze HT-SELEX data and are specifically designed to leverage particular properties of aptamers and the SELEX process.

These algorithms engage with the analysis process at the earliest possible moment by providing importing capabilities and quality control of raw sequencing data through AptaPLEX, a multithreaded demultiplexer and parser for HT-SELEX data.^12^ Compared to generic demultiplexers, AptaPLEX utilizes the primary structure configuration of aptamer reads to increase the total number of recovered oligonucleotides and effectively partition the raw data into the corresponding selection cycles based on barcoding information contained within the reads.

Alternatively, by invoking our SELEX simulator AptaSIM, sensible recreations of the selection process, incorporating features such as error-prone amplification, target affine selection, and aliquotation of the pool after each round, can be created and explored with AptaSuite. This in turn allows for the analysis of higher-order relationships regarding the selection pressures governing a particular experimental setup.^8^

A common first analysis step, after the initial data import, consists of grouping the sequences into aptamer families related to each other by primary structure, which evolved due to a combination of (hyper)mutagenic amplification followed by subsequent selection. To efficiently perform this clustering, and to trace the evolution of the resulting families throughout the selection, we have ported our previously published approach AptaCLUSTER into this new framework.^8^ AptaCLUSTER leverages the constant size of the randomized region in conjunction with locality sensitive hashing to outperform traditional clustering algorithms, which rely on expensive all-versus-all comparison techniques for their operations. This, and its multi-threaded architecture therefore enable clustering of datasets stemming from next-generation (and future) sequencing technologies in a computationally tractable manner. Building on the results of AptaCLUSTER, we additionally provide the ability to perform an in-depth analysis of the mutational landscape within aptamer families via AptaMUT,^8^ based on a theoretic model capable of discriminating favorable mutants from those that decrease the binding affinity to the target.

Another challenge paramount to the analysis of *in vitro* selection data centers on the identification of sequence-structure patterns shared among target-affine aptamers, which are responsible for the binding interaction between the species and target. In AptaSuite, this analysis is made possible through the integration of AptaTRACE,^13^ our computational approach that leverages the experimental design of the HT-SELEX protocol, RNA secondary structure information, and the potential presence of many secondary motifs to identify sequence-structure elements that show a signature of selection.

Finally, every aspect of the data and analysis results can be exported back to file in well-established formats for further downstream processing using third-party pipelines.

**Figure 2:**
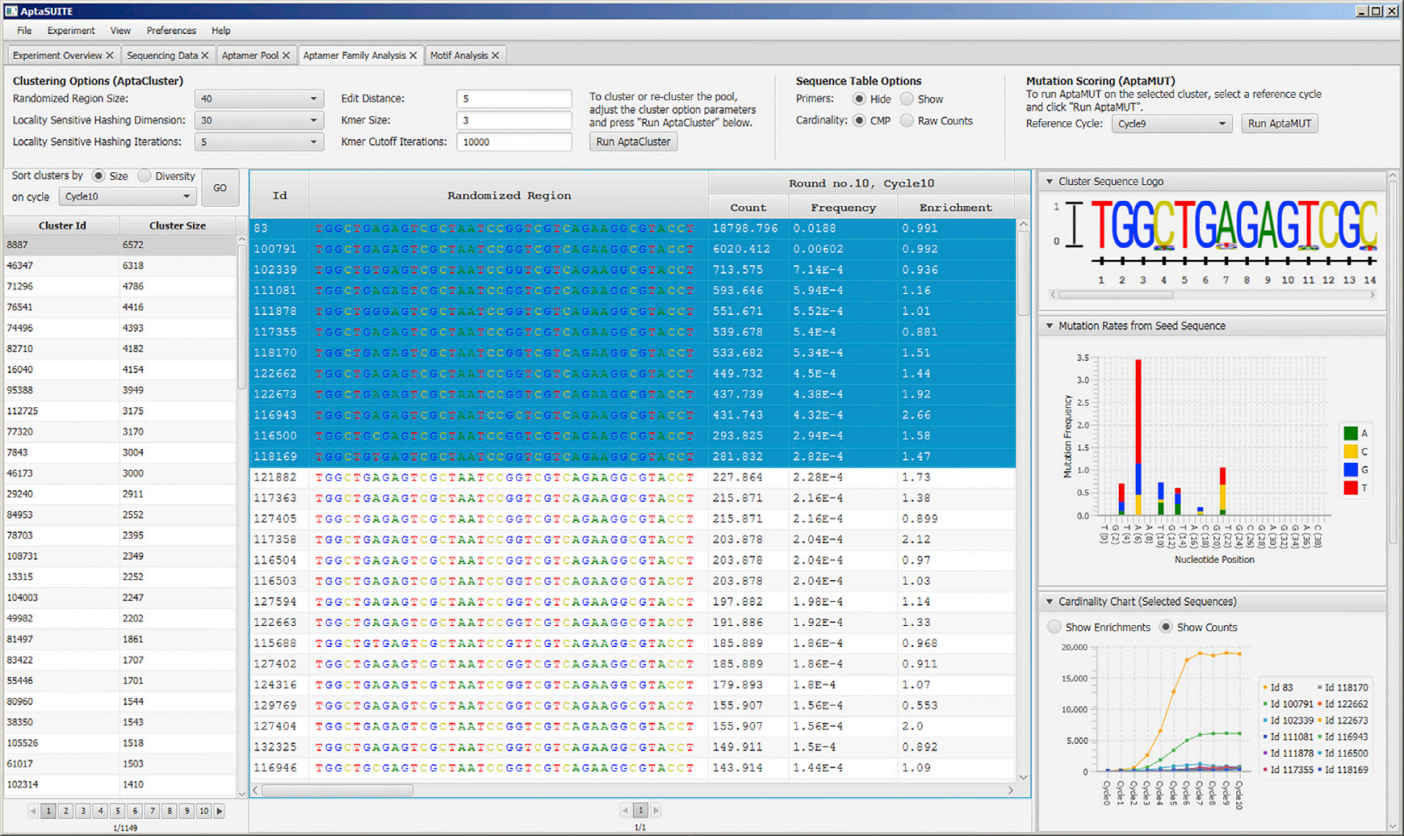
Screen Capture of the Graphical User Interface Screen capture of the graphical user interface in AptaSuite using data created with AptaSIM. Shown are the results of applying AptaCLUSTER onto the pool. This particular screen allows for the exploration of the identified aptamer families and their properties.

To maximize the applicability of AptaSuite to as many use cases as possible, every feature of our approach is made available to the user as both command line interfaces (CLIs) and graphical user interfaces (GUIs). The former can additionally be chained together in a variety of combinations to create seamless data processing pipelines. This allows AptaSuite to be deployed as fully automated applications in high-performance computing (HPC) environments, as point-and-click solutions for small- to mid-sized datasets on modern desktop hardware, or as a combination of the two, in which the heavy computation is outsourced to HPC systems whereas visualization and interpretation of the results can be performed locally using the graphical user interface (Figure 2).

This flexibility of AptaSuite is mainly attributed to our solution being implemented in pure Java without requiring any third-party dependencies, making this software truly platform independent and portable across a large array of hardware environments. In addition, AptaSuite is designed to be highly scalable with both, data size and CPU count, while minimizing the memory footprint by providing fast, off-heap data structures and storage solutions. Finally, its modular design and well-documented APIs allow for trivial extension with new algorithmic solutions as research in the field of *in vitro* selection progresses and novel questions calling for computer-aided problem solving arise.

To the best of our knowledge, AptaSuite represents the most comprehensive data engine to store and retrieve, manipulate, and analyze aptamer data to date. Our software is already being used by the aptamer community at large, and we are confident that AptaSuite will continue to establish itself as one of the de facto *in silico* analysis tools in the field of oligonucleotide *in vitro* selection.

### Availability

AptaSuite is an open source and the precompiled binaries, source code, and manual are available at https://github.com/drivenbyentropy/aptasuite.
